# Change in genetic size of small-closed populations: Lessons from a domestic mammal population

**DOI:** 10.1590/s1415-47572010000400011

**Published:** 2010-12-01

**Authors:** Farhad Ghafouri-Kesbi

**Affiliations:** Department of Animal Breeding and Genetics, Animal Science Research Institute of Iran, KarajIran

**Keywords:** pedigree, effective size, genetic drift, genetic diversity, genetic similarity, sheep

## Abstract

The aim of this study was to monitor changes in genetic size of a small-closed population of Iranian Zandi sheep, by using pedigree information from animals born between 1991 and 2005. The genetic size was assessed by using measures based on the probability of identity-by-descend of genes (coancestry, *f*, and effective population size, *N*_*e*_ ), as well as measures based on probability of gene origin (effective number of founders, *f*_*e*_ , effective number of founder genomes, *f*_*g*_ , and effective number of non-founder genomes, *f*_*ne*_ ). Average coancestry, or the degree of genetic similarity of individuals, increased from 0.81% to 1.44% during the period 1993 to 2005, at the same time that *N*_*e*_ decreased from 263 to 93. The observed trend for *f*_*e*_ was irregular throughout the experiment in a way that *f*_*e*_ was 68, 87, 77, 92, and 80 in 1993, 1996, 1999, 2002, and 2005, respectively. Simultaneously, *f*_*g*_ , the most informative effective number, decreased from 61 to 35. The index of genetic diversity (GD) which was obtained from estimates of *f*_*g*_ , decreased about 2% throughout the period studied. In addition, a noticeable reduction was observed in the estimates of *f*_*ne*_ from 595 in 1993 to 61 in 2005. The higher than 1 ratio of *f*_*e*_ to *f*_*g*_ indicated the presence of bottlenecks and genetic drift in the development of this population of Zandi sheep. From 1993 to 1999, *f*_*ne*_ was much higher than *f*_*e*_ , thereby indicating that with respect to loss of genetic diversity, the unequal contribution of founders was more important than the random genetic drift in non-founder generations. Subsequently, random genetic drift in non-founder generations was the major reason for *f*_*e*_ > *f*_*ne*_ . The minimization of average coancestry in new reproductive individuals was recommended as a means of preserving the population against a further loss in genetic diversity.

## Introduction

The long term survival of a population depends on the maintenance of sufficient genetic variation for individual fitness and population adaptability (Lacy *et al.*, 1995). In the wild, low genetic variation among individuals indicates the inability of a population to adapt to a changing environment and food supply, disease, or climatic conditions, or, in other words, the evolutionary flexibility of a population diminishes in parallel with the loss in genetic diversity (Lacy, 1995). In the context of animal breeding it has been widely recognized that increases in homozygosity often lead to lower viability and fecundity (inbreeding depression; Falconer, 1989). Moreover, low genetic variation among individuals limits the success of genetic improvement schemes (Gutiérrez and Goyache, 2005).

In managed populations, selection is the main factor responsible for the loss in genetic diversity. Whereas genetic drift is expected to occur in populations undergoing selection, Hedrick (2000) reported that in populations which are subjected to selection regimes, changes in allelic frequency are determined primarily by selection rather than random genetic drift. Where only a few superior animals are chosen and allowed to contribute to the gene pool of the next generation, a genetic bottleneck is imposed on the population as a whole. A direct consequence is a decrease in allelic variation, thereby limiting the response to selection, because long-term response is more dependent upon the alleles present in the population than on initial frequencies or heterozygosity (Allendorf, 1986).

For theoretically ideal populations, the loss of heterozygosity is inversely proportional to population size, for the smaller this is, the greater the loss in genetic diversity. Nevertheless, most real populations violate one or more of the assumptions for ideal populations (Funk *et al.*, 1999). Population genetics theory developed for ideal populations can be extended to real populations by computing effective population size (*N*_*e*_; Wright, 1931) which adjusts the actual number of active breeding animals to a sex ratio of 1:1. The effective population size is the size of an ideal population that has the same rate of inbreeding (or coalescence) as the real population under consideration. *N*_*e*_ is a measure of ‘identity-by-descent' (IBD) of genes, and determines the level of inbreeding, as well as the degree of genetic variation lost from populations due to random genetic drift. It has been frequently shown that *N*_*e*_ is often much less than the census size of the population (Scribner *et al.*, 1997; Hauser *et al.*, 2002; Turner *et al.*, 2002). In populations which undergo selection, either artificially or naturally, many individuals contribute little or nothing to the gene pool of future generations. In such a situation, active management is essential to assure that the genetically effective population size is not appreciably smaller than the recorded one (Foose *et al.*, 1986).

The estimation of parameters based on identity-by-descent of genes, such as *N*_*e*_, is considerably sensitive to the quality of pedigree information. Consequently, another approach, ‘the analysis of the probabilities of gene origin', which was first introduced by Dickson and Lush (1933) and further developed by MacCluer *et al.* (1986) and Lacy (1989), was recommended to assess genetic diversity. An important advantage of parameters which are obtained thereby is their being less sensitive to pedigree completeness when compared to parameters based on identity-by-descent of genes (Boichard *et al.*, 1997). In this technique, genetic diversity in a given population is assessed by measuring the genetic contributions of the founder animals. Since knowledge of the total number of founders is insufficient to ascertain the genetic basis of the population, owing to unknown pedigrees and the unequal contribution of founders to the genetic composition of the following generation, Lacy (1989) proposed the concept of effective number of founders, thereby accounting for the unequal contribution of founders and the idea of founder genome equivalents (also called effective number of founder genomes), as well as bottlenecks and random loss of alleles due to genetic drift. Caballero and Toro (2000) found that effective population size, effective number of founders and founder genome equivalents are interrelated in terms of coancestry and variance of contributions from ancestors to descendants, and proposed a new parameter, viz. the effective number of non-founder genomes, to describe the relationship between the effective number of founders and founder genome equivalents.

Only when accurate records have been kept can information on the genetic size of a population be obtained by analysing pedigree information. Pedigree analysis allows the population manager to assess the genetic structure of the population and to plan appropriate breeding strategies aimed at making a balance between genetic response and the loss of genetic diversity. In Iran, the conservation of animal genetic resources has received increasing attention over recent years. To my knowledge, this study is the first attempt in Iran to evaluate genetic diversity in a domestic mammal population using novel criteria of genetic diversity. In the current study, I analysed pedigree information of the animals registered in the herdbook of the Zandi sheep breeding station from 1991 to 2005, in order to evaluate changes in genetic size of the population during the experiment.

## Material and Methods

###  Animals and pedigree information

Zandi sheep constitute an Iranian small-sized breed, well adapted to the central semi-arid region of the country. Historically, this breed had its origin in the southern province of Iran known as Fars. In the late 1980s, an experimental flock was established in the Khojir national park between Tehran and Abali at 35°45' E and 51°40' N, 1547 m above mean sea-level, with temperate summers and cool winters, and an average rainfall of approximately 300 mm/yr. The aim was to establish a nucleus source for improving other flocks in the region. The founder animals (with unknown pedigrees) were purchased from various sheep farms in the region of the breeding station. In general, the flock was reared by following conventional industrial procedures. The mating season commences in August. Ewes on heat undergo artificial insemination (AI), with the restriction that mating between very closely related animals is avoided. The maximum number of ewes allocated to each AI ram is no more than 25-head per breeding year. Animals that do not conceive by AI are allocated to natural servicing. In this case, ewes are assigned to ram-breeding groups with an average mating ratio of 10-15 ewes per ram. Lambing commences in December. Coat-color in new-born is black, but gradually changes with age, in such a way that black, light-brown and gray adult animals are to be found.

Pedigree information of the tagged individuals has been recorded since 1991, thereby providing an opportunity to study the genetic structure of the population. The analysed pedigree consisted of 6035 animals (3283 males and 2752 females). Analysis involved populations born every three years from 1991 to 2005. Table 1 provides information about the population data used in the current study.

###  Methods of measuring diversity

#### Coancestry, *f*

Coancestry (Malécot, 1948) is defined as the probability that any two alleles, sampled at random (one from each individual), are identical copies of an ancestral allele.

#### The effective number of population size, *N*_*e*_

This parameter, obtained according to Gutiérrez *et al.* (2008) and in the form proposed by Gutiérrez *et al.* (2009) based on the individual increase in inbreeding (Δ*F*_*i*_). Δ*F*_*i*_ coefficients are computed as 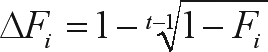, where *F*_*i*_ is the individual coefficient of inbreeding and *t* is the equivalent complete generations (Maignel *et al.*, 1996). This estimate of effective population size ( ${\bar{N}}_{e}$), denominated ‘realized effective size' by Cervantes *et al.* (2008), can be computed from ΔF, which, in turn, can be computed by averaging the Δ*F*_*i*_s of the *n* individuals included in a given reference subpopulation, as ${\bar{N}}_{e}=1/2\bar{\Delta F}$.

#### The effective number of founders, *f_e_*

The parameter *f*_*e*_ indicates the number of equally contributing founders that would produce the same level of genetic diversity as that observed in the current population. Lacy (1989) estimated the effective number of founders as being 
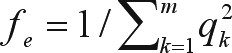
, where *q*_*k*_ is the expected proportional genetic contribution of founder *k*, calculated by the average relationship of the founder to each animal in the current population, whereas *m* is the total number of founders.

#### The effective number of founder genomes, *f*_*g*_

The effective number of founder genomes indicates how many founders would be required to produce the same genetic diversity as actually found in the population, if all contributed equally and no lost of alleles occurred (Lacy, 1989). According to Caballero and Toro (2000), parameter *f*_*g*_ was obtained by the inverse of twice the average coancestry of the individuals included in a pre-defined reference population. Estimates of *f*_*g*_ are of value for estimating genetic diversity in reference subpopulations relative to a base line. The degree of genetic diversity (GD) in the reference population relative to the base population is approximated as: GD = 1 - 1/2*f*_*g*_ (Lacy, 1989; 1995), when genetic diversity is expressed with the ‘expected hetrozygosity' (Nei, 1973).

#### The effective number of non-founder genomes, *f*_*ne*_

The fourth type of effective number of animals, the effective number of non-founder genomes, only accounts for the effect of genetic drift in non-founder generations. This effective number is obtained as *f*_*ne*_^-1^ = *f*_*g*_^-1^- *f*_*e*_^-1^ (Caballero and Toro, 2000).

Genealogical analyses on pedigree information were carried out using the ENDOG, v. 4.6 program (Gutiérrez and Goyache, 2005).

## Results

**Figure 1:**
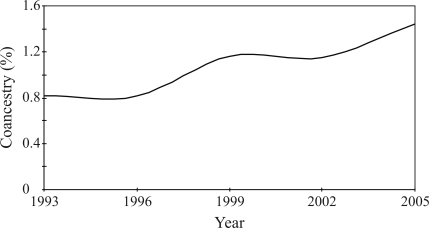
Evolution of coancestry during the analysed period.

**Figure 2:**
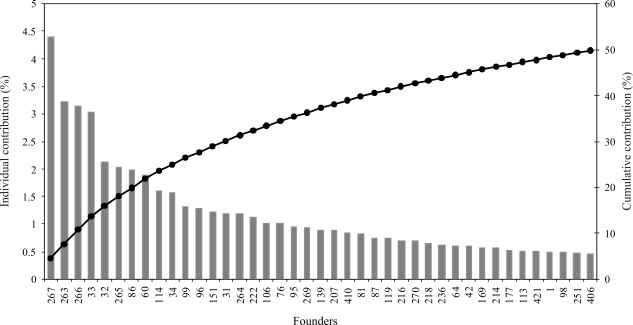
Individual and cumulative genetic contribution of the most influential founders. The contribution of the 41 most influential founders represents 50% of the total genetic diversity.

The evolution in coancestry (*f*) in the population studied during the experiment is shown in Figure 1. The average coancestry increased significantly (p < 0.01) throughout the experiment from 0.81% in 1993 to 1.44% in 2005. The estimates of *N*_*e*_, *f*_*e*_, *f*_*g*_, *f*_*ne*_, and GD are shown in Table 2. The change in *N*_*e*_ was non-significant (p > 0.05), although there was a decrease from 263 to 93 during the analysed period. Likewise, *f*_*e*_ showed a non-significant trend (p > 0.05) during the experiment and was 68, 87, 77, 92 and 80 in 1993, 1996, 1999, 2002 and 2005, respectively. In contrast there was a significant decrease (p < 0.01) in both *f*_*g*_ and *f*_*ne*_. For *f*_*g*_, this being from 61 in 1993 to 35 in 2005, with a reduction of approximately 2% in the corresponding diversity index (GD), and for *f*_*ne*_ from 595 in 1993 to 61 in 2005. The ratio of *f*_*e*_ to *f*_*g*_ increased significantly (p < 0.01) from 1.11 to 2.28 during the period. Individual and cumulative genetic contributions of the first 41 most influential founders in the population studied appear in Figure 2. As shown, they presented 50% of total genetic diversity. The unequal contribution of these 41 founders to the current gene pool are also highlighted.

## Discussion

It has been argued that coancestry is a more informative parameter than other measures of diversity, since it contains all currently available information on the future rate of inbreeding. The average coancestry of animals in a population forecasts the average coefficient of inbreeding in the subsequent generation. For this reason, coancestry has been utilized to calculate the expected future effective population size (Sørensen *et al.*, 2005). Coancestry highlights the degree of genetic similarity of individuals in a breeding population. High coancestry means low diversity, with less ability in selecting better animals, as the difference between individuals is narrower. Although, coancestry is an informative parameter, and has been shown to be useful for conservation purposes (Toro *et al.*, 2002), as this is a population-level measurement, the number of animals included in the data under analysis, as well as the depth of pedigree included, impact upon its estimation.

The concept ‘effective population size', introduced by Sewall Wright, is fundamental in population genetics, and is considered a key parameter in conservation genetics, because *N*_*e*_ is instrumental for indicating not only the degree of genetic drift, but also population viability (Frankham *et al.*, 2002). Simply put, the lower *N*_*e*_, the higher will be the probability for a population to go extinction. Despite the increase in adult population census-size from 223 in 1993 to 481 in 2005, the size of the effective population decreased during the analysed period, thereby indicating that, parallel to an increase in adult population census-size, average relationship between individuals also increased through kin mating, followed by a higher level of inbreeding and, in consequence, a smaller *N*_*e*_. The required minimal *N*_*e*_ has been the subject of much research. Rasch and Herrendorfer (1990; in FAO, 1992), based on a literature survey, recommended an *N*_*e*_ of 200 for maintaining a genetically constant population over 50 generations. Brem *et al.* (1990; in FAO, 1992) reported that a population is not threatened when the effective population size is over 50, and where there is a minimum of 10 males. Nevertheless, for effective selection, an *N*_*e*_ of at least 100 is necessary (FAO, 1992). Except in 1993, when *N*_*e*_ was 263, estimates of *N*_*e*_ were below 100. Thus breeders should be concerned about controlling inbreeding in their respective breeding programs.

The effective number of founders (*f*_*e*_) depends on both their total number (*m*), as well as the disequilibrium among their expected contributions to the gene pool. If all the founders contribute equally, then *f*_*e*_ = *m*, but when this is not so then *f*_*e*_ < *m*. As shown in Figure 2, of the total genetic variability (100%) in the population, 50% could be attributed to 41 founders. Influential founders, as indicated when estimating *f*_*e*_, are those with a high contribution to the current population. They may have a considerable impact on the trait being selected, if they possess beneficial alleles for the selected trait to pass on to their progeny. In populations with minimum inbreeding, the effective number of founders is expected to be one-half of effective population size. Other situations (*f*_*e*_ ≥ *N*_*e*_) indicate that the breeding structure has undergone certain changes since the founder generation (Sørensen *et al.*, 2005). While *f*_*e*_ takes into account the unequal founder contributions, in populations which have undergone a bottleneck, the effective number of founders is overestimated, since the effect of such possible bottlenecks is not taken into consideration, especially in animal breeding, where selection for specialized types of animals imposes strong bottlenecks on the populations. Moreover, there is another important handicap when calculating *f*_*e*_, brought about by the phenomenon, ‘converged genetic contribution of founders' (Bijma and Woolliams, 1999; Caballero and Toro, 2000). When random mating is practiced in a closed population and assuming there is no genetic drift, the contribution of founders (*q*_*k*_'s) will be very similar, whereupon *f*_*e*_ will approach a relatively constant value within a few generations, seeing that founder contributions will remain stable. Even in the presence of selection, *f*_*e*_ will approach a constant value as long as new founders are not added to the population. Caballero and Toro (2000) mathematically formulated this phenomenon and illustrated that after a few generations, variation in founder contribution will approach an asymptotic state, whereupon, *f*_*e*_ will also become constant, without further change. In simulations of true random mating, this will happen to a large degree within five generations, although, in real life, populations tend to undergo a certain degree of positive assortative mating which slows this down (J.A. Woolliams, personal communication). Thus, a management program based on the maximization of *f*_*e*_, while partially effective in the initial generations, will be completely ineffective thereafter.

The most informative effective number is that of founder genomes (*f*_g_), which, besides dealing with the total loss in diversity, is directly related to genetic diversity itself. As shown by Lacy (1995), the relationship between the current population's mean coancestry and *f*_*g*_ is *f*_*g*_ = (1/2*c*), where *c* is the mean coancestry of all the individuals in the current population, including the relationship of each individual to itself. The value shows that as the population becomes more related, as would happen with any closed population, *f*_*g*_ decreases. A significant decrease in *f*_g_ reveals a reduction in the genetic size of the population as regards founder genes. As expected, during the experiment, the decrease in estimates of *f*_*g*_ was accompanied by a drop in the corresponding GD index which comprises all the causes of reduction in genetic diversity. It is noteworthy that while *f*_*g*_ is the more accurate description of the amount of founder variation present in a population, it does not account for mutation or migration, and, if mutations do occur, a slightly higher amount of heterozygosity could be present.

The ratio of the effective number of founders to the effective number of founder genomes, which was higher than 1 throughout the experiment, indicated the presence of bottlenecks and genetic drift in the development of this population of Zandi sheep.

Last effective number, viz. effective number of non-founder genomes, accounts only for the effect of genetic drift in non-founder generations. By using *f*_*ne*_, it is possible to clarify which, as regards the loss of genetic diversity, is the more important, random genetic drift accumulated in non-founder generations or the unequal contribution of founders. Where *f*_*e*_ > *f*_*ne*_, the reduction in genetic diversity is more related to genetic drift accumulated in non-founder generations, whereas if *f*_*ne*_ > *f*_*e*_, the unequal contribution of founders is the major reason for the loss of genetic diversity. In our population and during the period 1993 to 1999, *f*_*ne*_ was much larger than *f*_*e*_, thereby indicating unequal contribution of founders to be the more important. Afterward, random genetic drift in non-founder generations was the major reason for the loss of genetic diversity as *f*_*e*_ > *f*_*ne*_.

In general, the results obtained here show that in small-closed populations, especially those undergoing managed selection, genetic diversity can be lost at a rapid rate, with two important consequences, first, the loss in heterozygosity, followed by inbreeding depression, and second the loss of allelic variants, thereby limiting long-term responses to selection. Although inbreeding could not be avoided or indefinitely limited, care must be taken in minimizing its rate of occurrence. Various methods have been proposed to minimize the loss of genetic diversity. The minimization of group coancestry has proved to be the most efficient method to conserve genetic diversity (Meuwissen, 1997; Caballero and Toro, 2000). Minimization of average coancestry of the new reproductive individuals could be implemented in the population in order to preserve its genetic variability. Therefore, this strategy should be incorporated in breeding schemes where animals are selected based on phenotypic values, thereafter continuing the process based on minimum coancestry to avoid any further decrease in the genetic size of the population.

## Figures and Tables

**Table 1:** Description of the Zandi sheep population.

No. of animals in the pedigree file	6035
No. of males	3283
No. of females	2752
No. of animals with progeny	1647
No. of animals without progeny	4391
No. of animals with both parents unknown	451
No. of animals with one parent unknown	904
Maximum contribution of a founder to the current gene pool	4.4%
Minimum contribution of a founder to the current gene pool	0.008%
Average contribution of founders to the current gene pool	0.07%

**Table 2:** Changes in measures of genetic diversity during the analysed period^a^.

Year	${\bar{N}}_{e}$	*f*_*e*_	*f*_*g*_	*f*_*e*_/*f*_*g*_	*f*_*ne*_	GD
1993	263	68	61	1.11	595	0.99
1996	88	87	61	1.42	332	0.99
1999	48	77	43	1.79	93	0.99
2002	50	92	42	2.13	81	0.99
2005	93	80	35	2.28	61	0.98

${\bar{N}}_{e}$, effective population size; *f*_*e*_, effective number of founders, *f*_*g*_, effective number of founder genomes; *f*_*ne*_, effective number of non-founder genomes; GD, genetic diversity index.
